# PTEN Tumor-Suppressor: The Dam of Stemness in Cancer

**DOI:** 10.3390/cancers11081076

**Published:** 2019-07-30

**Authors:** Francesca Luongo, Francesca Colonna, Federica Calapà, Sara Vitale, Micol E. Fiori, Ruggero De Maria

**Affiliations:** 1 Istituto di Patologia Generale, Università Cattolica del Sacro Cuore, Largo Francesco Vito 1, 00168 Rome, Italy; 2 Department of Oncology and Molecular Medicine, Istituto Superiore di Sanità, 00161 Rome, Italy; 3 Scientific Vice-Direction, Fondazione Policlinico Universitario “A. Gemelli”–I.R.C.C.S., Largo Francesco Vito 1-8, 00168 Rome, Italy

**Keywords:** cancer stem cells, PTEN, therapy resistance, targeted therapy

## Abstract

*PTEN* is one of the most frequently inactivated tumor suppressor genes in cancer. Loss or variation in *PTEN* gene/protein levels is commonly observed in a broad spectrum of human cancers, while germline *PTEN* mutations cause inherited syndromes that lead to increased risk of tumors. *PTEN* restrains tumorigenesis through different mechanisms ranging from phosphatase-dependent and independent activities, subcellular localization and protein interaction, modulating a broad array of cellular functions including growth, proliferation, survival, DNA repair, and cell motility. The main target of *PTEN* phosphatase activity is one of the most significant cell growth and pro-survival signaling pathway in cancer: PI3K/AKT/mTOR. Several shreds of evidence shed light on the critical role of *PTEN* in normal and cancer stem cells (CSCs) homeostasis, with its loss fostering the CSC compartment in both solid and hematologic malignancies. CSCs are responsible for tumor propagation, metastatic spread, resistance to therapy, and relapse. Thus, understanding how alterations of *PTEN* levels affect CSC hallmarks could be crucial for the development of successful therapeutic approaches. Here, we discuss the most significant findings on *PTEN*-mediated control of CSC state. We aim to unravel the role of *PTEN* in the regulation of key mechanisms specific for CSCs, such as self-renewal, quiescence/cell cycle, Epithelial-to-Mesenchymal-Transition (EMT), with a particular focus on *PTEN*-based therapy resistance mechanisms and their exploitation for novel therapeutic approaches in cancer treatment.

## 1. Introduction

First identified in 1997, *PTEN* (Phosphatase and TENsin homolog deleted on chromosome 10) is among the most commonly mutated tumor suppressor genes in human cancers [1,2,3,4] (Table 1). *PTEN* is a negative regulator of the phosphatidylinositol-3-kinase (PI3K)/AKT cascade, which controls cell growth, proliferation, survival, and metabolism. The fine regulation of this axis represents a critical node of oncogenic transformation [5].

*PTEN* is a haploinsufficient tumor-suppressor gene since the loss of 50% of its function compromises tumor suppression [6,7]. Further, even subtle variations of *PTEN* function can have dramatic effects on cancer predisposition and tumorigenesis, suggesting a strict dosage-dependency of *PTEN* tumor suppression [8]. The apparent paradox that complete ablation of *PTEN* can be detrimental to tumor growth, in the absence of other mutations, is due to the activation of p53-dependent senescence that counteracts tumor progression (obligate haploinsufficiency) [9]. Genetic ablation of *PTEN* leads to the development of different tumor types in mice [10,11,12]. In humans, germline mutations of *PTEN* cause a group of rare autosomal dominant syndromes (*PTEN* hamartoma tumor syndromes, PHTS), characterized by increased risk for specific malignancies (breast, thyroid, renal, and endometrial cancers) and neurodevelopmental disorders, such as autism and mental retardation [13,14,15]. In somatic cancers, including glioblastoma (GBM), endometrial, breast and prostate cancer, *PTEN* function can be compromised by a variety of mechanisms. In addition to genetic inactivation, *PTEN* expression is tightly controlled both at transcriptional and post-transcriptional level [5]. Epigenetic inhibitory mechanisms involving *PTEN* promoter hyper-methylation and histone acetylation were reported in several cancers [16,17,18,19,20]. Moreover, multiple transcription factors control *PTEN* expression and a number of microRNAs (miRNAs, miRs) were identified as *PTEN* suppressors. Interestingly, post-translational modifications including ubiquitination, phosphorylation, acetylation, oxidation and sumoylation or interaction with other proteins affecting the activity and subcellular localization of *PTEN* are often associated with tumorigenesis [5,21,22,23]. Furthermore, severe *PTEN* deficiency is associated with advanced tumor stage (70% in glioblastomas and 60% in advanced prostate cancers) and resistance to therapy, especially in targeted therapies for the receptor tyrosine kinases (RTKs) pathway (e.g., trastuzumab) [24]. Increasing evidence indicates *PTEN* as a prognostic and predictive biomarker for drug response in several tumors (e.g., prostate, breast, endometrium) [25]. Altogether, the multi-level control of *PTEN* expression depicts a highly dynamic landscape in which even subtle fluctuations of this protein can induce very significant effects.

*PTEN* is expressed early during embryonic development and ubiquitously in the adult [11]. The main *PTEN* catalytic activity is the lipid phosphatase [5,26]. *PTEN* dephosphorylates phosphatidylinositol (3,4,5)-trisphosphate (PIP_3_) to phosphatidylinositol-4,5-bisphosphate (PIP_2_) [26]; PIP_3_ is generated through the phosphorylation of the 3-position of the PIP_2_ inositol ring by PI3K, a lipid kinase activated by RTKs and G-protein coupled receptors (GPCRs) [27]. The PI3K complex activates the 3-phosphoinositide-dependent kinase 1 (PDK1) and AKT [24]. Consequently, *PTEN* antagonizes the PI3K signaling [26,28] and the downstream activation of PDK1/AKT and AKT/mammalian target of rapamycin (mTOR) [27,29,30]. Through the inhibition of the oncogenic and pro-survival PI3K/AKT/mTOR axis, *PTEN* acts as a tumor suppressor by regulating transcription, translation, cell cycle progression, induction of cell death, stimulation of angiogenesis, and stem cell self-renewal [5]. In addition, this axis is an essential target for anti-cancer agents, especially in tumors displaying elevated activity of the mTOR pathway [31].

*PTEN* exerts a dual protein-phosphatase activity, targeting both phospho-serine/threonine and phospho-tyrosine substrates [5,32]. Besides the self-regulation (by auto-dephosphorylation), *PTEN* controls the activity of several target proteins as glycogen synthase kinase-3 (GSK3), BAD, Caspase 9, I*κ*B, focal adhesion kinase 1, cAMP-responsive element-binding protein 1 (CREB1), proto-oncogene tyrosine-protein kinase SRC, insulin receptor substrate 1 (IRS1), modulating pivotal biological functions including inhibition of cell migration, invasion, cycle arrest and survival [5,33,34,35]. Interestingly, *PTEN* plays also a non-enzymatic role, behaving as a scaffold protein in both the nucleus and the cytoplasm [5]. Although the primary subcellular localization of *PTEN* is cytoplasmic and membrane-bound, *PTEN* protein can translocate into the nucleus where it controls critical cellular functions like genomic stability, pre-mRNA alternative splicing, apoptosis, cell cycle arrest and senescence [36,37]. These nuclear activities of *PTEN* are mainly lipid-phosphatase-independent [38,39]. *PTEN* nuclear localization occurs preferentially in primary, differentiated, and resting cells rather than cancer cell lines, underlining how the nuclear pool is critical for the tumor suppressive functions of *PTEN* [40,41,42,43,44]. Consistently, the absence of nuclear *PTEN* is associated with more aggressive cancers. These observations pave the road for the use of *PTEN* as a prognostic marker. Moreover, *PTEN* can also be secreted and transferred to neighboring cells, packaged and exported within exosomes. Upon transfer in target cells *PTEN* localizes in the cytoplasm and mitochondria, where it can regulate mitochondrial function and energy production, or in the nucleolus, where it inhibits ribosomal biogenesis [45,46,47,48]. Recent studies demonstrated the existence of a translational variant, named *PTEN*-Long (*PTEN*-L), characterized by an additional 173aa-domain at the N-terminal containing a secretion signal that enables this membrane-permeable isoform to be secreted and uptaken by recipient cells in a paracrine manner [46]. *PTEN*-L is expressed in normal tissues and detectable in human serum and plasma whereas lower expression is reported in human breast tumor and mouse models of glioblastoma [49]. Of note, administration of *PTEN*-L protein to tumor-bearing mice showed an interesting therapeutic potential [46]. Moreover, Liang and collaborators reported an N-terminally extended form, named *PTEN*-α, localized in the cytoplasm and mitochondria, where it can regulate mitochondrial function and energy production [47]. Further, another variant named *PTEN*-β, was recently identified in the nucleolus, where it binds to and dephosphorylates nucleolin, thus inhibiting ribosomal DNA transcription and ribosomal biogenesis [48]. Strategies focused at improving *PTEN* secretion and uptake by target cells may offer a feasible way for tumor treatment [5,50].

In addition to the intrinsic onco-suppressive function of *PTEN*, several studies underline the pivotal role of *PTEN* in the modulation of the tumor microenvironment, acting at different levels on cancer cells, stromal compartment, and immune response, thus controlling disease initiation, progression and metastases. *PTEN* loss in mammary stromal fibroblasts induces an oncogenic secretome that modifies the tumor microenvironment by promoting tumor angiogenesis, extracellular matrix (ECM) deposition, and macrophage recruitment, accelerating malignant transformation [51]. Moreover, a *PTEN*-null stroma can increase the expansion of mammary epithelial stem cells (MaSC), influencing the mammary stem cell niche [52]. In accordance, the simultaneous ablation of *PTEN* and Src homology phosphatase 2 (Shp2) in the hepatic microenvironment promotes the earlier expansion of liver tumor-initiating cells (TIC) and enhances tumorigenesis [53]. Further, *PTEN*-null senescent prostate cancer creates an immunosuppressive tumor microenvironment through cytokines secretion and recruitment of myeloid-derived suppressor cells [54]. Of note, another study revealed the plasticity of *PTEN* expression in the “co-evolution” between metastatic cells and the stroma: the unique brain metastatic microenvironment induces tumor cells to lose *PTEN* expression by astrocyte-derived exosomal miRNA targeting *PTEN*, promoting metastatic outgrowth [55].

Finally, despite the well-defined anti-tumoral function of *PTEN* in the greatest majority of cancers, in some specific contexts a pro-tumoral role of *PTEN* has been documented. Specifically, in pre B acute lymphoblastic leukemia (ALL), a cancer of immature B cells, *PTEN* ablation unexpectedly prevents malignant transformation: loss of one or both alleles of *PTEN* induces pre-B ALL cells death and is able to clear transplanted recipient mice of leukemia [56]. In line with these data, it was shown that *PTEN* can acquire tumor-promoting functions by enhancing the stability of gain-of-function p53 mutants in glioma cells, both *in vitro* and *in vivo* [57]. Another novel gain-of-function *PTEN* mutation, frequently observed in gliomas, is the truncation of the C-terminal region (*PTEN*Δ51) that shifts *PTEN* towards tumor promoting activity [58].

The term Cancer Stem Cells (CSCs) defines a subpopulation of multipotent cells with unique properties of self-renewal and long-term clonal maintenance, responsible for tumor progression, metastatic spread, recurrence and therapeutic resistance [59,60]. This nomenclature is based on the common properties that these cells share with normal stem cells, behaving as a reservoir for cancer. However, the definition of stemness applied to the complex tumor context relies on peculiar parameters that do not strictly stick to classical concepts [61].

Although CSCs do not necessarily coincide with the “cell of origin” of the tumor, they are endowed with and defined by the capacity to sustain serial xenograft formation in mice. This consideration has led to the interchangeable use of different designations, as CSCs and TICs. Given the capacity to foster tumor propagation and spread, these cells have also been referred to as Tumor Propagating Cells (TPCs). For ease of reading, we adopt throughout this review the term CSCs to refer to cancer cells endowed with stem-like phenotypes, tumorigenic potential and therapy resistance properties.

CSCs were first described in acute myeloid leukemia, where the clear hierarchical organization of stem/progenitor/precursor cells reflects the pattern observed in normal hematopoiesis. Subsequently, putative CSCs were identified in multiple solid tumors, including melanoma, brain, breast, colon, lung, and prostate cancers at variable frequencies across different tumor types (2–40%) [62,63]. Several groups proposed the isolation of CSCs based on the expression of specific markers such as CD34, CD38, CD44, CD24, CD90, CD133, aldehyde dehydrogenase 1, Hoechst 33342 dye exclusion [64] and/or by the capacity to grow in selective media [65,66]. However, cancer cell stemness is better defined by functional assays, such as the ability to self-renew and differentiate, giving rise to a tumor reproducing the patient’s pathology and propagating in serial transplantation experiments [67]. Furthermore, CSCs exhibit multiple treatment resistance features, including upregulation of drug-efflux pumps, a superior DNA-repair capacity, enhanced protection against ROS, quiescent state, immune evasion [68].

Recent advances allowed genetic-lineage tracing experiments that enabled the *in-situ* identification and tracking of stem cells in solid tissues/tumors. These studies revealed discrepancies with the models derived by transplantation-based approaches, possibly because the potential of a given cell type does not necessarily correspond to the real behavior in the original environment. Interestingly, lineage-tracing and other studies have pointed out the concept of ‘cancer stem state’, where transit-amplifying cells can reversibly acquire the stem cell attributes in response to epigenetic and microenvironmental factors [59,68,69,70,71]. This CSC feature, named ‘plasticity’, enhances the complexity of intratumor heterogeneity and urges the understanding of the crosstalk between tumor and non-tumor cells as well as the identification of key players responsible for the transition into the stem cell status, which would facilitate the identification of therapeutic strategies able to eradicate cancer.

Reportedly, several pathways exerting a pivotal role in the control of normal stem cell self-renewal and differentiation are altered in CSCs, such as Wnt, Hedgehog, and Notch [72,73]. Given the central function of *PTEN* in the regulation of these and other pathways controlling stem cell maintenance, homeostasis, self-renewal and migration, as well as the crosstalk with tumor microenvironment, the modulation of this tumor suppressor is deeply involved in the biology of CSCs [49,74]. In this review, we will discuss the current advances in *PTEN*-mediated regulation of CSC hallmarks, with a particular focus on self-renewal, quiescence, EMT/migration, survival, and therapy resistance.

## 2. *PTEN*-Mediated Control of CSCs Hallmarks

### 2.1. PTEN and Self-Renewal

Self-renewal is a specific feature of stem cells, consisting of controlled division (symmetric or asymmetric) able to maintain the undifferentiated state to the progenies, thus indefinitely perpetuating the stem cell pool [119]. In a variety of malignancies, the ability to alter normal self-renewal pathways allows CSCs to drive malignant transformation [120]. *PTEN*, p53 and several pathways such as Notch, Wnt, and Hedgehog regulate the self-renewal of healthy stem cells. Deregulation of these pathways promotes stem cell expansion and increased spheroid-forming ability *in vitro* with the consequent acquisition of a high tumorigenic potential [121]. Loss of *PTEN* in normal stem cells contributes to malignant transformation in several tissues. Conditional deletion of *PTEN* in adult hematopoietic stem cells [HSCs] triggers leukemia onset in mice, while alterations of *PTEN* functions in bronchioalveolar and neural stem cells are involved in increasing their expansion and neoplastic potential [122,123,124].

In cancer cells, several data pointed out the role of *PTEN* in the control of self-renewal, measured *in vitro* as sphere-forming activity: *PTEN* mutant cells display high tumor-sphere formation capacity and maintenance upon serial passages in glioma, prostate, and breast cancer models [74,125,126,127]. Moreover, the mutation of one or both alleles of *PTEN* is associated with the successful generation of proliferating neurosphere cultures from glioblastoma surgical specimens, suggesting a higher capacity to self-renew [128].

*In vivo*, *PTEN* deletion promotes the expansion of CSCs and enhances their ability to form tumors capable of serial passaging in NOD/SCID mice. Serial dilution experiments in mice demonstrated that *PTEN* knockout cells display a higher tumorigenic potential/CSCs content compared to wild type cells, in models of breast, prostate and lung cancers [126,127,129]. Moreover, both in normal and malignant mammary epithelial cells, *PTEN* knockdown fosters the CSC compartment, through the generation of hyperplasic lesions and contributing to cancer onset in humanized mice [127].

Interestingly, the concomitant loss of *PTEN* and p53 promotes an undifferentiated status in neural stem cells and increases their proliferation and self-renewal through C-MYC regulation [130]. Consistently, in primary prostate epithelial cells depleted for both *PTEN* and *P53*, a higher self-renewal capacity compared to wild type primary cells was reported. Accordingly, CRE-mediated depletion of *PTEN* and *P53* in normal mouse prostate epithelial cells generates highly tumorigenic cells, containing a stable sub-population endowed with self-renewal and multi-lineage differentiation capacities [131,132].

**Figure 1:**
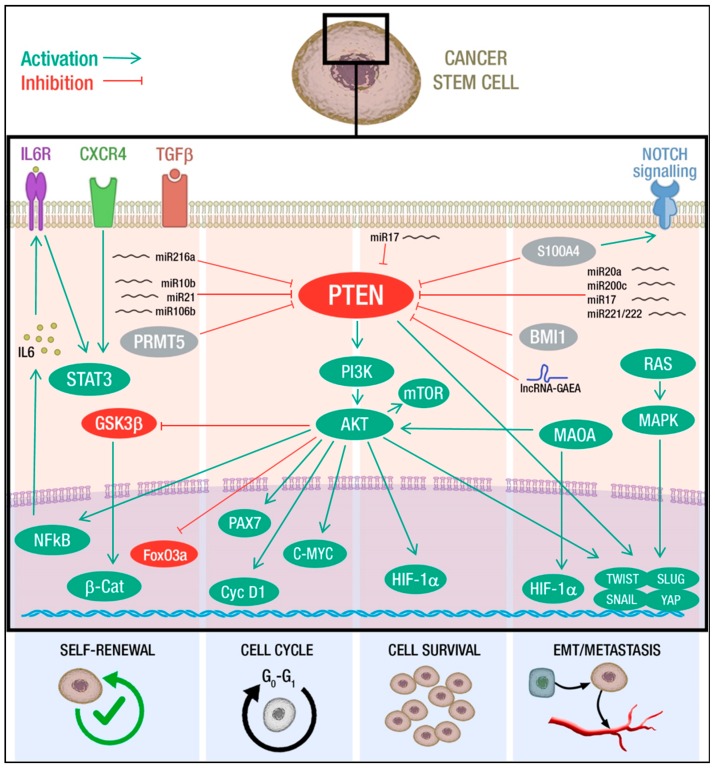
Mechanisms of PTEN-mediated control of cancer stem cells (CSCs) hallmarks. PTEN deficiency promotes self-renewal through inhibitory phosphorylation of FoxO3a [126,136] and GSK3β, which increases nuclear β-catenin localization [127]. Furthermore, it induces STAT3 activation by NFkB-mediated IL6 transcription [129] and CXCR4 expression [135]. MicroRNAs (miR-10b [140], miR-21 [133], miR-106b [139] and TGFβ-induced miR-216a [136]) and PRMT5-mediated methylation [141] are also involved in the downregulation of PTEN and regulation of self-renewal. Hyperactive AKT acts on cell cycle stimulating proliferation trough PAX7 [124], cyclinD1 [142] or C-MYC [143]. PTEN loss may trigger G0 cell cycle arrest and quiescence of CSCs [122,142,144,145,146,147,148,149]. Activation of mTOR is required for CSC survival and can be associated with the activation of collateral pro-survival pathways such as HIF-1α [150]. S100A4 [151] and BMI1 [134], along with several miRNAs (miR-20a/miR-200c [152], miR-17, miR-221/222 [153]) impair PTEN function, thus promoting EMT and metastatic progression. The lncRNA-GAEA inhibits PTEN lipid phosphatase activity, switching on its protein phosphatase activity and promoting the accumulation of EMT master regulators such as TWIST, SNAIL, and YAP [154]. Moreover, activation of the RAS/MAPK pathway [155], NOTCH1 signaling [156], and MAOA [157] cooperate with PTEN/PI3K/AKT axis to promote the EMT program.

Mechanistically, *PTEN* loss promotes self-renewal through the activation of PI3K/AKT signaling, which, in turn, modulates several downstream pathways. Indeed, the inhibition of PI3K/AKT/mTOR activity by specific inhibitors leads to a decrease in the CSC population in nasopharyngeal carcinoma [133], glioma [74] pancreatic carcinoma [134], lung cancer [129,135], prostate cancer [126,131] and breast cancer [127]. Besides the involvement of the PI3K/AKT axis, the AKT-mediated phosphorylation of FOXO3a and GSK-3β increases the nuclear β-catenin localization, which potentiates tumorigenicity through the maintenance of stem-like cells populations and clonogenic potential [126,127,136]. Wnt/β-catenin and PTEN/PI3K/AKT pathways also collaborate to promote self-renewal induced by Aryl hydrocarbon receptor and the cytochrome P450 1A1 (CYP1A1) activation [137]. Further, abnormal activation of PI3K/AKT/mTOR pathway increases chemokine receptor type 4 (CXCR4) expression and, in turn, promotes the maintenance of stemness through STAT signaling [135]. STAT3 hyper-activation can also result from the activity of the PI3K/AKT/IL-6 axis: AKT induces IkB degradation, which allows NF-kB to enter the nucleus and activate IL-6 transcription, able to promote self-renewal and tumorigenesis [129,138] (Figure 1). Upstream of *PTEN*, several mediators of self-renewal have been identified. As discussed, *PTEN* expression can be modulated at post-transcriptional level by miRNAs. TGF β-mediated deregulation of miR-216a in cancer, as well as upregulation of miR-106b, miR-10b, and miR-21, affects *PTEN* levels and thus CSC self-renewal [133,136,139,140]. Interestingly, a recent work on glioblastoma primary cells highlighted a new level of PTEN expression control, focusing on the role of the protein arginine methyltransferase-5 (PRMT5) on self-renewal and proliferation of neurospheres. This report shows that *PTEN* is a target of PRMT5 in the context of primary GBM neurospheres, but not in the differentiated counterparts and that *PTEN* downregulation, upon methylation by PRMT5, is required for stem cells maintenance through the AKT/ERK axis [141].

### 2.2. PTEN and Cell Cycle

Another hallmark of CSCs is the ability to reversibly enter a state of slow proliferation and/or quiescence, with cells resting in G0 phase, but ready to promptly reactivate the cell cycle upon external stimuli [69]. Quiescence is an actively regulated status observed also in a subset of adult tissue specific stem cells, where it was defined and characterized. In cancer, quiescent CSCs are the driving force for relapse and therapy-resistance. Among the main determinants of quiescence are p53, Rb, cyclin-dependent kinase inhibitors, paired box protein 7 (PAX7), Notch signalling. Interestingly, quiescent cells are under the control of post-transcriptional mechanisms of gene expression regulation, with miRNAs playing a central role as underlined by the preferential use of distal polyadenylation sites, generating long 3′UTRs exposed to miRNA binding. Relying on post-transcriptional regulation allows a rapid switch toward active cycling upon proper stimuli. This is further accomplished by the maintenance of key genes in a heterochromatic status that is permissive for transcription, with RNA Polymerase II (RNA PolII) stuck on specific promoters, ready to activate the elongation phase and switch on proliferative programs [158].

*PTEN* plays a crucial role in the regulation of the quiescence and the cell cycle entry of both normal and transformed stem cells (SCs), with *PTEN* loss often fostering the cancerous phenotype. Yilmaz and colleagues took advantage of a mouse model for leukemia to show that *PTEN* deletion leads to the exhaustion of the normal stem compartment in favor of leukemic cancer stem cell (L-CSC) expansion and acute leukemia development [122,144]. Interestingly, treatment with rapamycin is sufficient to restore the normal HSCs pool, suggesting the involvement of mTOR protein downstream of *PTEN* and envisaging the chance to selectively target L-CSCs without consequence for the normal HSCs [122]. The same mechanism occurs in BCR-ABL chronic myeloid leukemia (CML) where an increased percentage of dividing HSCs was found in *PTEN* deficient mice with overt leukemia, thus indicating that *PTEN* governs the transition between the quiescent and activated state of HSCs to maintain their physiologic pool. Consistently, *PTEN* expression induces cell cycle arrest in BCR-ABL expressing leukemia cells [142,144,159,160]. Mechanistic insights came from muscle stem cells, where *PTEN* loss induces AKT phosphorylation and by that FOXO1 cytoplasmic translocation and inhibition of NOTCH pathway, resulting in quiescent stem cells exhaustion [161].

Of note, mouse models harboring the brain-specific deletion of *PTEN* provided evidence on the role of *PTEN* in restricting neural stem cell proliferation and size [162]. Specifically, loss of *PTEN* in mouse brain determines enhanced G0 cell cycle exit and self-renewal capacity [163]. An interesting study defined the transforming capacity of *PTEN* loss in neural stem cells (NSCs). Mouse NSCs derived from both WT and *PTEN*^−/−^ ESCs were assayed for tumorigenic potential, both *in vitro* and *in vivo*, clarifying that *PTEN* loss acts through PAX7 activation to induce transcriptional and metabolic changes leading to neoplastic transformation [124]. Moreover, the activation of PTEN/mTOR/STAT3 pathway in breast cancer cells is required for the viability and maintenance of a subpopulation enriched in CSCs, defined as the side population (SP). These findings suggest that the SP compartment contains a significant number of quiescent cells, providing a possible explanation for drug resistance [145,146,147]. Similar evidence aroused from studies on glioblastoma CD133^+^ stem-like SP population, comprising the majority of cells in G0/G1 phase [148,149].

The role of *PTEN* in cell cycle control of CSCs is not limited to quiescence regulation. Peng and colleagues showed that PI3K/AKT pathway is involved in cyclin D1 activation, maintaining cells at G1 stage. In *PTEN* deficient acute myeloid leukemia (AML) mice, a high number of cyclin D1-expressing cells populated the bone marrow. Furthermore, after rapamycin administration, L-CSCs were depleted and normal HSCs restored in *PTEN*-deficient AML mice, providing evidence of PI3K/mTOR pathway involvement [142]. Of note, in contrast to other hematologic malignancies, in *PTEN*-null T-cell acute lymphatic leukemia (T-ALL) L-CSCs are not in a quiescent state, but rather are actively cycling. This feature is consistent with the overexpression of C-MYC that fuels the cell cycle entry of L-CSCs. C-MYC overexpression is a common feature of *PTEN*-null T-ALL and is due to *Tcrα/δ-c-Myc* translocation but also results from *PTEN* deletion and consequent enhancement mTOR activity [143] (Figure 1).

### 2.3. PTEN and Cell Survival 

Cell survival is a key mechanism for tumor persistence and represents a critical node for the therapeutic outcome. In addition to PI3K/mTOR/AKT axis hyperactivation, *PTEN* loss influences other pathways involved in cell survival. Li and colleagues showed that *PTEN* negatively regulates *PTEN*/HIF-1α angiogenic pathway in glioblastoma. In particular, they observed that under stress conditions such as starvation or chemotherapeutic drug treatment, suppression of *PTEN* by miR-17 activates HIF-1α, thus promoting survival, migration, and angiogenesis. Furthermore, in GBM cells, HIF-1α overexpression contributes to the generation of tumor stem-like cells endowed with increased capacity to form colonies and neurospheres and marked drug-resistance [150] (Figure 1).

*PTEN* loss can induce cellular senescence (*PTEN*-loss-Induced Cellular Senescence, PICS). Although the partial loss of *PTEN* determines an increases in proliferation, its biallelic inactivation, prevalent in advanced stages of cancers [4,5,164,165], leads to cellular senescence, a protective mechanism to restrict tumorigenesis that is dependent on p53 and may follow mTORC1-mediated increased translation and stabilization [164,166]. Abou-Kehir and colleagues, using a *PTEN*/*P53* null prostate cancer mouse model, explained the oncogenic effect of these double mutations with the absence of TP53-dependent cellular senescence after *PTEN* loss [131]. Moreover, the function of *PTEN* in senescence could be mediated by other players. For instance, an interesting study evaluated head and neck squamous-cell carcinoma (HNSCC) development under PTEN and TGFβ loss of signaling. In this model system, *PTEN* deletion is not sufficient for epithelial cells transformation and HNSCC onset, due to the induction of AKT- and p53-dependent senescence. In this context, mutation of TGFβR1 triggers the evasion of senescence and expansion of cancer stem cells. Indeed, *TGFβR1/PTEN* double conditional knockout (2cKO) mice show significantly increased malignant lesions with enhanced stem cell features and reduced apoptosis [167].

### 2.4. PTEN and EMT-Metastasis

Neoplastic cells can reversibly enter the CSC status through the activation of the Epithelial-to-Mesenchymal-Transition (EMT), an *epigenetic* program which drives cells to lose many of their epithelial attributes and switch to mesenchymal characteristics [168]. Plasticity of CSCs sustains the fluctuation between franc epithelial and mesenchymal programs, generating a gradient of intermediate conditions that determine intra-tumor heterogeneity. CSCs endowed with mesenchymal features gain the ability to disseminate to other tissues and establish new metastatic foci. Conversely, once the metastatic niche is colonized, CSCs revert to a more epithelial status and are able to propagate and generate secondary tumors. This complex transition is controlled by cell intrinsic mechanisms as well as by microenvironmental stimuli. *PTEN* loss or downregulation correlates with the acquisition of EMT traits. In lung cancer, hypoxic microenvironment determines an unbalance in the phospho-PTEN (pPTEN)/PTEN ratio that is associated with EMT [169]. Further, TGFβ-induced phosphorylation of PTEN C-terminal domain determines a reduced phosphatase activity that mediates EMT induction in lung cancer cells [170].

Several factors regulate EMT process, migration, and invasion acting upstream of PTEN. It was reported that knockdown of oncogenic BMI-1 (B-lymphoma Moloney murine leukemia virus insertion region-1) and S100A4 (S100 calcium binding protein A4), involved in EMT and invasion ability, decreases phosho-AKT (pAKT) and increases PTEN expression in pancreatic and head-neck CSCs respectively, highlighting the link of PI3K/AKT pathway to EMT process [134,151]. Furthermore, miR-20a, miR-200c, and miR-221/222 regulate PI3K/AKT pathway targeting *PTEN*, thus modulating self-renewal capabilities, invasion, and metastasis cascade through EMT process in ovarian and breast CSCs [152,153]. Oncogenic miR-17 promotes cell motility and invasion through *PTEN* suppression and subsequent HIF1α and VEGF up-regulation in glioblastoma [150]. In prostate cancer patients with elevated Gleason score, high Monoamine oxidase (MAOA) expression correlates with low *PTEN* expression. Wu and colleagues observed that MAOA overexpression increases HIF1α stabilization, activates the EMT-promoting genes AKT/FOXO1 and VEGF-A, thus enhancing TWIST1 expression. Subsequently, Liao and colleagues took advantage of a prostate-specific *PTEN*/*MAOA* knockout mouse model and observed a decreased incidence of invasive prostate adenocarcinoma development associated with reduced AKT activity, attenuated CSC markers and reduced clonogenic capacity [157,171].

Recent work has elucidated the role of the long-non-coding RNA *G*lucose *A*roused for *E*MT *A*ctivation (lncRNA-GAEA) on PTEN enzymatic activity. In particular, lncRNA-GEAE, induced by high glucose levels, could drive poly-ubiquitination of PTEN, thus inhibiting its lipid phosphatase while activating the protein phosphatase activity. This switch determines the dephosphorylation and consequent accumulation of EMT master regulators, such as TWIST, SNAIL, and YAP1 [154].

Moreover, Mulholland and colleagues highlighted how alteration of RAS/MAPK signaling potentiates *PTEN*/PI3K/AKT axis effects, cooperating to promote EMT and drive primary tumor and metastatic prostate cancer progression. In a context of *PTEN* loss and Ras activation, the authors observed an enhanced expression of EMT markers in stem/progenitor cells *in vitro*, and metastasis development in orthotopic transplantation models [155].

A recent report showed that Connexin43 (Cx43) regulates *PTEN* protein phosphatase activity, reducing migration and invasion abilities of glioblastoma stem cells (GSCs). Mechanistically, Cx43 inhibits c-Src activity and upregulates *PTEN* expression, which contributes to FAK inactivation by dephosphorylation [172].

Collectively, these findings sustain the role of *PTEN* in the control of several cancer stem cells hallmarks such as self-renewal, quiescence, motility, and invasiveness, all features consistent with the positive association between *PTEN* loss and poor prognosis and metastasis (Figure 1). In particular, *PTEN* has a determinant role in the reversible acquisition of specific CSC traits, as quiescence and EMT, thus controlling plasticity and by that the capacity to overcome stressful conditions as hypoxia and nutrient deprivation or resist to therapeutic insults.

## 3. *PTEN* Implication in Therapy Resistance

**Figure 2:**
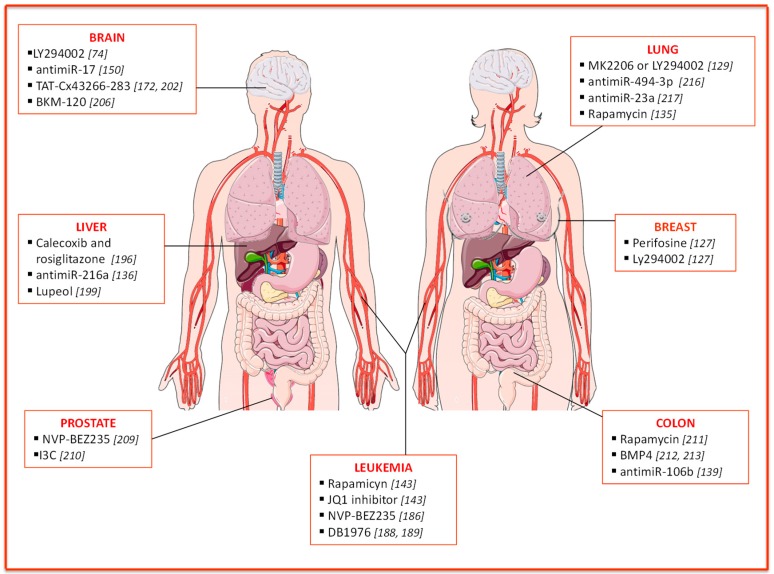
Targeting PI3K/PTEN/AKT/mTOR pathways for cancer therapy. Schematic depicting of possible strategies/drugs to overcome PTEN-mediated resistance to conventional therapy in several malignancies. **Nervous system cancer**: specific PI3K/AKT inhibitors, LY294002 and BKM-120, in combination with conventional chemotherapy, inhibit Glioblastoma Multiforme (GBM) and Sonic Hedgehog Medulloblastoma (SHH-MB) CSCs, respectively. Anti-miR-17 and TAT-Cx43266-283, by mimicking the effects of Connexin 43 (CX43), increase PTEN expression and inhibit glioma stem cells tumorigenic features. **Liver cancer**: treatment with Celecoxib (cyclooxygenase-2 (COX-2) inhibitor) and rosiglitazone reduce AKT phosphorylation and increase PTEN protein levels, thus affecting cell proliferation. Anti-miR-216a and lupeol, a phytochemical compound, decrease the stem population through PTEN modulation. **Prostate cancer**: PI3K-mTOR dual inhibitor, NVP-BEZ235, in combination with conventional chemotherapy, leads to significant tumor regression in mice, targeting both prostate cancer progenitors (PCPs) and bulk tumor. I3C (indol-3-carbinol), a derivative of cruciferous vegetables, is able to restore PTEN activity leading to suppression of tumorigenesis. **Leukemia**: mTOR inhibitor rapamicyn, PI3K-mTOR dual inhibitor, NVP-BEZ235, JQ1 inhibitor targeting c-Myc pathway, and DB1976, a compound that disrupts the interactions between SPI1 and its targets, all result in a significant reduction of leukemic stem cells (L-CSCs). **Colon cancer**: treatment with rapamicyn, in combination with chemotherapy, inhibits tumor growth. Treatment with anti-miR-106b may overcome radio-resistance in colon cancer. BMP4 (Bone Morphogenetic Protein 4) inhibits PI3K/AKT pathway through PTEN up-regulation. **Breast cancer**: treatment with PI3K/AKT inhibitors, LY294002 and perifosine, alone or in combination with chemotherapy, reduces mammary stem cell population and tumor growth in mice. **Lung cancer**: treatment with LY294002, MK2206, and rapamicyn inhibits PI3K/AKT/mTOR pathway acting on the maintenance of lung stem cells through chemokine receptor modulation. Anti-miR-494-3p prevents metastasis and tumor progression while anti-miR-23a is able to upregulate PTEN expression, restoring lung cancer stem cells (LCSCs) sensitivity to chemotherapy.

The CSC features mentioned above, together with enhanced DNA repair and efflux pump activities, confer intrinsic resistance to drugs on this cell population. The PI3K/AKT/PTEN/mTOR pathway plays a role in multiple mechanisms of chemo- and radio-resistance, suggesting that targeting of this pathway could be a promising strategy to restore drug sensitivity in CSCs and improve patients’ survival [173]. Here we will discuss PTEN-based resistance mechanisms and possible strategies to modulate PTEN-dependent pathways with the attempt to overcome resistance (Figure 2).

### 3.1. Breast Cancer

Despite the success of trastuzumab-based therapy in HER2+ breast cancer treatment, the occurrence of resistance remains significant and strongly affects the overall efficacy of the current therapeutic regimens [174,175]. Indeed, about 15% of women treated with trastuzumab face metastatic disease within the first year [176,177] and the majority of patients develop drug resistance after one to two year of treatment [178,179]. Trastuzumab-based therapy targets multiple pathways downstream of HER2 including PI3K and MAPK [180,181]. The first hints proving the role of PTEN in trastuzumab resistance came in 2004 when Nagata and colleagues observed activation of PTEN upon trastuzumab treatment and showed that PTEN is required for the anti-tumor efficacy of trastuzumab, since its inhibition is sufficient to induce resistance [182]. Accordingly, an elegant approach exploiting a short hairpin RNA (shRNA) library demonstrated that PTEN is the mediator of resistance. The authors showed that *PTEN* loss, together with the more frequent mutation in *PIK3CA*, can be used as a biomarker with a substantial predictive value for therapy sensitivity and patients’ outcome [183].

As discussed above, *PTEN* knockdown determines the enrichment of normal and malignant mammary stem cells and triggers a significant increase in AKT phosphorylation levels, leading to GSK3β-dependent β-catenin activation and fueling malignant transformation. Consistently, treatment with perifosine (AKT inhibitor) or Ly294002 (PI3K inhibitor), alone or in combination with chemotherapy, reduces mammary stem cell population and tumor growth in breast cancer xenografts [127].

Recent evidence proposes NOTCH-1 as the main actor in PTEN-mediated resistance to trastuzumab. Baker and colleagues reported the presence of NOTCH-1 modulation together with *PTEN* down-regulation in HER2^+^ breast cancer resistant cells. They showed that NOTCH-1 induced the inhibition of PTEN through the activation of ERK1/2, thus promoting survival and self-renewal of trastuzumab-resistant breast CSCs [176]. In accordance with these data, recent studies highlighted that continued use of trastuzumab in HER2+ breast cancer, with loss of PTEN function, induces the epithelial-mesenchymal transition (EMT) and transforms HER2+ to a more aggressive triple negative breast cancer cell type, endowed with increased proliferation and metastatic potential *in vivo* [178,179].

### 3.2. Leukemia

Hematopoietic stem cells (HSCs) are the first and best-characterized adult stem cells. Normal hematopoiesis requires specific interactions between the bone marrow niche and HSCs, and perturbation of the microenvironment components may contribute to the generation of leukemic stem cells. L-CSCs are refractory to therapy and eventually sustain resistance and relapse. As already mentioned, alterations in the PTEN pathway are commonly associated with leukemogenesis [144,184]. Interestingly, the inactivation of PTEN leads to increased AKT phosphorylation and mTOR activation, which can be reversed with the specific mTOR inhibitor, rapamycin [185]. In *PTEN*-null mice, rapamycin is able to restore the normal HSC self-renewal capacity, suggesting that *PTEN*-null HSCs exhaustion can result from increased mTOR activation [122,160].

Schubbert and colleagues proposed a combination therapy to selectively eliminate L-CSCs, targeting both the PI3K and C-MYC pathway (rapamycin and JQ1 inhibitor) [143]. Of note, replacing rapamycin, with NVP-BEZ235, a dual inhibitor of PI3K and mTOR, resulted in cell cycle arrest and reduced L-CSCs compartment [186]. Interestingly, the identification of a novel regulatory circuit, responsible for leukemia ‘stemness’ maintenance, paved the road for a new approach to hit L-CSCs. Specifically, in a background of several driver mutations, including *PTEN* loss, the SPI1/PU.1 transcription factor was identified as a master regulator of L-CSCs stemness properties [187]. Treatment of *PTEN*-null T-ALL leukemic mice with DB1976 (a compound that disrupts the interactions between SPI1 and its targets), alone or in combination with rapamycin, significantly reduces the number of L-CSCs [188,189].

### 3.3. Liver Cancer 

Hepatocellular carcinoma (HCC) accounts for about 70–85% of liver cancers, and it is one of the most common malignancies worldwide [190]. Reportedly, the upregulation of Cyclooxygenase-2 (COX-2), an inducible enzyme frequently found in inflammatory tissue, correlates with angiogenesis, invasion, relapse, chemo-resistance, and tumorigenesis in HCC [191], ultimately promoting hepatoma cell growth by inhibiting apoptosis through AKT activation [192,193]. Inhibition of AKT signaling leads to decreased hepatic CSCs biomarkers, CD133, and CD44 [194,195]. Celecoxib, a selective COX-2 inhibitor, reduces AKT phosphorylation and induces growth inhibition and apoptosis in HCC cells, stimulating peroxisome proliferator-activated receptor gamma (PPARy) as well as PTEN. Treatment with rosiglitazone, a PPARγ agonist, determines an increase in endogenous and celecoxib-induced PTEN protein levels in hepatoma cells, which in turn contributes to the inhibition of hepatic cancer stemness and growth through the reduction of AKT activation and CD133 and CD44 expression levels [196].

Furthermore, *PTEN* mutant mice are enriched in liver CSCs (CD133+ CD45-) [197]. Previous studies demonstrated the role of TGFβ in hepatic CSCs generation: TGFβ-exposed cells show elevated levels of phosphorylated AKT and suppression of PTEN expression [198]. Among *PTEN*-targeting microRNAs, miR-216a is upregulated in HCC, and its specific inhibition can rescue *PTEN* expression and suppress CSC self-renewal capacity [136].

An interesting study showed that lupeol, a phytochemical compound, decreases liver cancer stem population through *PTEN* upregulation. *In vivo*, lupeol treatment suppresses tumor growth and synergistically cooperates with standard chemotherapy acting on both the PTEN-AKT pathway and the ATP-binding cassette super-family G member 2 (ABCG2) [199].

### 3.4. Brain Cancer

Glioblastoma multiforme (GBM) is the most malignant form of glioma with a 5-year survival rate of 5% [150]. Besides surgery, the current therapeutic regimen is based on radiotherapy and temozolomide (TMZ) administration. *PTEN* loss is extremely frequent in GBM (40%) and correlates with TMZ resistance [200]. Consistently, treatment with TMZ doubles the fraction of stem-like SP cells, thus enhancing their tumorigenic potential. Inhibition of PI3K/AKT pathway with a specific inhibitor (LY294002) has been proposed to reduce multi-drug resistance mediated by ABCG2 in cancer stem-like cells and improve GBM chemosensitivity [74].

As already mentioned, the increased expression of miR-17 and consequent down-regulation of *PTEN* fuel the tumorigenic features in glioma stem cells (GSCs). Of note, miR-17 overexpression confers drug resistance in docetaxel-treated GSCs by regulating PTEN/HIF-1α pathway [150]. Connexin43 (CX43) is an integral membrane protein, weakly expressed in GSCs [201]. Cell-penetrating peptides containing the CX43 residues 266–283 (TAT-Cx43266-283) can mimic the effects of CX43 [202]. Interestingly, by inhibiting c-Src and increasing PTEN, these peptides reduce FAK (focal adhesion kinase) activity and lead to inhibition of migration and invasion in GSCs [172].

Medulloblastoma (MB) is an aggressive pediatric cerebellar tumor [203], displaying a perivascular niche enriched in CD133+ stem cells, involved in radiotherapy resistance. Specifically, a single dose of radiation induces p53-dependent apoptosis in the tumor bulk and transient cell cycle arrest in resistant stem cells, which survive due to PTEN malfunction and activation of the PI3K/AKT/mTOR pathway. Interestingly, the elucidation of PTEN involvement in this mechanism of resistance suggests the use of AKT inhibitors in the clinic to increase the efficacy of radiotherapy [204].

About 50% of Sonic hedgehog (SHH)-MB are characterized by *PTEN* loss, which correlates with worse overall survival [205]. Singh and colleagues validated the therapeutic efficacy of PI3K inhibitors (BKM-120) in the treatment of SHH-MB stem cells, suggesting a promising combination of PI3K inhibitors and chemotherapy (cisplatinum and TMZ) for the treatment of this disease [206].

### 3.5. Prostate Cancer

Conventional therapies for prostate cancer include surgery, hormonal-, radio- and chemo-therapy [207]. Despite the overall initial efficacy of these approaches, a large percentage of patients eventually relapse. The eradication of CSCs should be the primary goal to avoid resistance and relapse. *PTEN* is one of the most frequently mutated genes in prostate cancer and is often responsible for therapy resistance [208]. Therefore, the analysis of *PTEN* mutations might predict therapeutic response, while targeting of *PTEN* and related proteins holds promise for therapeutic efficacy [209]. Interestingly, in 2010 Dubrovska and colleagues showed how a combinatorial approach, based on conventional chemotherapy and NVP-BEZ235, a dual PI3K/mTOR inhibitor, leads to significant tumor regression targeting both bulk tumor cells and prostate cancer progenitors (PCPs), respectively.

Furthermore, in a recent study, Pandolfi and coworkers demonstrated that the indol-3-carbinol (I3C), a derivate of cruciferous vegetables, has potent anti-tumor properties. They provided evidence on the mechanism of action of this novel compound, showing that it strongly inhibits the ubiquitin E3 ligase WWP1, which acts downstream of MYC pathway and mediates the PTEN inactivation. Of note, I3C treatment can restore PTEN activity, leading to a potent suppression of tumorigenesis driven by the PI3K-AKT pathway in prostate tumor spheroids, organoids, and xenografts. These findings open a new perspective of a “reactivation” approach to tumor therapy [210].

### 3.6. Colon Cancer

The development of targeted therapies has brought to the clinic several molecules able to interfere with specific pathways. In colorectal cancer, the combined treatment of Trametinib and Everolimus, a MAPK/ERK pathway inhibitor and mTOR inhibitor, respectively, results in significant inhibition of tumor growth [211]. The identification of novel potential targets or therapeutic tools is essential to hit CSCs and overcome their innate resistance to conventional therapies. *PTEN* levels are usually very low in CSCs as compared to the more differentiated cells. We observed that bone morphogenetic protein 4 (BMP4) delivery and thymosine β4 targeting display anti-tumor activity in colorectal (CR) cancer by inducing CR-CSC differentiation and inhibition of PI3K/AKT pathway through the contribution of *PTEN* upregulation [212,213]. Moreover, recent evidence pointed out how miR-106b, which inversely correlates with *PTEN* levels, induces radio-resistance in colorectal cancer cells by enhancing tumor-initiating cell capacity [139].

### 3.7. Lung Cancer

Lung cancer is the leading cause of cancer-related death worldwide. Non-small cell lung cancer (NSCLC) is frequently characterized by an aberrant AKT activation arising from loss of *PTEN* or PIK3CA/AKT1 activating mutations. Epidermal Growth Factor Receptor Tyrosine Kinase Inhibitors (EGFR-TKi) represent the treatment of choice for EGFR-mutated NSCLC, but the occurrence of resistance has sharply limited their efficacy. In about 4% of cases, *PTEN* loss promotes EGFR-TKi resistance by inducing AKT activation and EGFR reactivation [214]. Several studies pointed out the involvement of PTEN and downstream pathways in CSCs survival and resistance mechanisms. For instance, the activation of PI3K/AKT pathway can promote human bronchial epithelial cells (BEAS-2B) tumorigenic potential, sustained by a subpopulation of CSCs. Furthermore, the treatment of NSCLC cells with specific AKT inhibitors (MK2206 or LY294002) strongly impairs AKT phosphorylation, and thus, the spheroid-forming capability of CSCs [129]. Moreover, PI3K/PTEN/AKT/mTOR pathway influences the maintenance of NSCLC stem cells through CXCR4 modulation. The expression of this chemokine receptor is associated with *PTEN* downregulation and pAKT upregulation in gefitinib-resistant A549 cell line (A549/GR) [215]. Treatment of A549/GR with the PI3K inhibitor LY294002 suppresses CXCR4 expression and spheroids formation, while restoring wild type *PTEN* reduces pAKT levels and CXCR4 expression. Conversely, in the presence of mutant *PTEN*, rapamycin treatment suppresses CXCR4 and CD133 expression, underlining the involvement of mTOR pathway downstream of PTEN [135]. Furthermore, in a model of murine lung carcinogenesis, the modulation of both NOTCH1 and PTEN/PI3K/AKT signaling through miR-494-3p correlated with lung CSCs (LCSCs) maintenance, cancer progression, and metastasis. Interestingly, miR-494-3p upregulation correlates with worse survival rates in a cohort of lung cancer patients, suggesting that this miRNA could be a new therapeutic target [216]. Notably, recent evidence suggests that miR-mediated regulation of *PTEN* is involved in resistance to anti-EGFR targeted inhibitor (erlotinib). Despite its ability to target EGFR, erlotinib treatment fails to inhibit PI3K and AKT activation in LCSCs. Han and colleagues reported a higher miR-23a expression in LCSCs that correlates with low *PTEN* expression. Of note, miR-23a inhibition via antisense oligonucleotides restores LCSCs sensitivity to erlotinib by upregulation of *PTEN*, suggesting their possible combined approach for NSCLC treatment [217].

## 4. Concluding Remarks

A wealth of studies has provided convincing evidence on the crucial role of PTEN function and modulation in human cancer susceptibility. Due to the pivotal role of PTEN in several key cellular functions, mutations in this tumor suppressor occur in a wide variety of tumors. The frequent loss/alteration of *PTEN* expression may be envisioned as a possible Achille’s heel for cancer, offering a common strategy to fight several different malignancies. A deeper understanding of the regulatory networks and the major alterations of *PTEN* expression and its related PI3K/AKT/mTOR pathway opens new insights for pharmacological targets and prognostic tumor biomarkers. Therapeutic strategies directed to activate or reactivate PTEN function represent a promising perspective for tumor treatment [210]. Moreover, PTEN regulates CSCs development and maintenance, specifically affecting critical features of these cells through downstream signaling pathways such as WNT, NOTCH, PI3K/AKT, MAPK and NF-kB (Figure 1). The plastic interconversion between CSCs and non-CSCs is not hardware-defined, but rather depends on multiple environmental cues. *PTEN* loss can influence the response to such stimuli at multiple levels, eliciting the transition to the cancer stem cells state by promoting EMT, quiescence, self-renewal and by that governing CSCs plasticity which is crucial for tumor progression, metastasis, and therapy resistance.

Thus, highlighting the complex network of PTEN within the CSCs population and the tumor niche microenvironment, may provide considerable information for the identification of successful therapeutic strategies aiming at overcoming CSC-mediated drug resistance and enhancing the tumor suppressive function of PTEN in cancer.

## Figures and Tables

**Table 1:** Incidence of *PTEN* alterations in diverse malignancies. The type of alteration is specified along with the relative % of patients displaying that specific alteration. Studies with more than 30 patients were included in the analysis.

Site	Malignancy Type	Molecular Mechanism(s) of *PTEN* Alteration and Incidence (%)	Reference
Prostate	Prostate cancer	Mutation: 12–26% LOH: 10–62% Reduced expression: 27–95%	[75,76,77,78,79,80,81]
Breast	Breast cancer	Mutation: <7% LOH: 29–63% Reduced expression: 8–55%	[82,83,84,85,86]
Brain	Glioma	Mutation: 12–44% LOH: 32–84% Reduced expression: 69%	[42,87,88,89]
Ovary	Ovarian carcinoma	Mutation: <9% LOH: 32–61% Reduced expression: 23–55%	[90,91,92,93,94]
Liver	Liver cancer	Mutation: <5% LOH: 27–79% Reduced expression: 30–63%	[95,96,97,98]
Lung	Non-small-cell lung cancer	Mutation: <5% LOH: 3–19% Reduced expression: 41–73%	[99,100,101,102]
Colorectum	Colorectal cancer	Mutation: 17–20% LOH: 20–30% Reduced expression: 12%	[103,104,105,106]
Blood	Myeloid leukemia	Deletion: rare LOH: Absent Reduced expression: 24%	[107,108]
	Lymphoid leukemia	Deletion: 8–63% LOH: NA Reduced expression: 6–17%	[109,110,111,112]
Head-neck	Head and neck squamous cell carcinoma	Deletion: 2–23% LOH: 41% Reduced expression: 31–60%	[113,114,115,116,117,118]
